# Tracking the mechanical dynamics of human embryonic stem cell chromatin

**DOI:** 10.1186/1756-8935-5-20

**Published:** 2012-12-21

**Authors:** Elizabeth Hinde, Francesco Cardarelli, Aaron Chen, Michelle Khine, Enrico Gratton

**Affiliations:** 1 Laboratory for Fluorescence Dynamics, Department of Biomedical Engineering, University of California, Irvine, CA 92697, USA; 2 Center for Nanotechnology Innovation @NEST, Istituto Italiano di Tecnologia, Pisa, Italy; 3 Department of Chemical Biochemical Engineering and Materials Sciences, University of California, Irvine, CA, 92697, USA; 4 Department of Biomedical Engineering, University of California, Irvine, CA, 92697, USA

## Abstract

**Background:**

A plastic chromatin structure has emerged as fundamental to the self-renewal and pluripotent capacity of embryonic stem (ES) cells. Direct measurement of chromatin dynamics *in vivo* is, however, challenging as high spatiotemporal resolution is required. Here, we present a new tracking-based method which can detect high frequency chromatin movement and quantify the mechanical dynamics of chromatin in live cells.

**Results:**

We use this method to study how the mechanical properties of chromatin movement in human embryonic stem cells (hESCs) are modulated spatiotemporally during differentiation into cardiomyocytes (CM). Notably, we find that pluripotency is associated with a highly discrete, energy-dependent frequency of chromatin movement that we refer to as a ‘breathing’ state. We find that this ‘breathing’ state is strictly dependent on the metabolic state of the cell and is progressively silenced during differentiation.

**Conclusions:**

We thus propose that the measured chromatin high frequency movements in hESCs may represent a hallmark of pluripotency and serve as a mechanism to maintain the genome in a transcriptionally accessible state. This is a result that could not have been observed without the high spatial and temporal resolution provided by this novel tracking method.

## Background

Embryonic stem cells (ESCs) are pluripotent cells that are derived from the inner cell mass of the pre-implantation embryo at the blastocyst stage. They are characterized by their potential to self-renew indefinitely and to differentiate into any of the three germ layers - endoderm, mesoderm and ectoderm [1]. This dual capacity places opposing constraints on the organization of the ESC genome [2]. Self-renewal requires that the ESC genome maintains a cellular memory that specifies its pluripotent capacity, whilst pluripotency relies on the ESC genome being in a highly plastic state so as to have the ability to enter any one distinct differentiation pathway [3]. How these two key functional properties of ESCs are maintained is largely unknown. However, from a structural point of view it is thought that the open conformation of ESC chromatin and hyperdynamic association of chromatin structural proteins are in part responsible [2,4-6].

Studies in several systems indicate that ESCs are rich in euchromatin and as differentiation progresses, undergo a rapid reorganization of large areas of the genome to accumulate highly condensed, transcriptionally inactive heterochromatin regions. For example, by transmission electron-microscopy, Park *et al*. proved the transition from fine granular chromatin in undifferentiated human ESCs (hESCs) to irregularly-shaped heterochromatic nuclei in retinoic acid-induced differentiating cells [7]. The number of heterochromatin foci has also been shown to increase during differentiation in embryonal teratocarcinoma F9 stem cells as well as in murine cells [8]. Consistent with these studies is the elevated expression level of several ATP-dependent chromatin-remodeling factors in ESCs [9], which if disrupted, can result in premature embryonic death prior to implantation [10-13]. Although informative, the conclusions drawn from all of these studies were based on fixed cells stained with a chromosome fluorescent marker or specific antibody for nuclear compartments; thus chromatin dynamics were not directly observed. This point is critical given the observation made by Meshorer *et al*. from live cell imaging, that stem cell chromatin actively ‘breathes’ between different structural conformations associated with hyperdynamic binding of structural proteins [2,4,5].

Methods for monitoring fast chromatin movement directly in living cells are thus crucial to advancing our understanding of the mechanism(s) by which chromatin structure maintains pluripotency. Direct measurement of chromatin dynamics *in vivo* is, however, challenging as high spatiotemporal resolution is required. In the past it has been achieved by use of a GFP-Lac-repressor protein that binds stably to an integrated modified Lac operator [14]. With this approach, chromatin was shown to undergo constrained Brownian motion confined to sub-regions of the nucleus [15]; a motion that seems to be highly influenced by the association of chromatin with nuclear compartments [16]. More recently, by 2-photon microscopy and single-particle tracking, Levi *et al*. revealed that chromatin-constrained diffusion was interrupted by ATP-dependent abrupt leaps of about 150 nm that last for around one second, demonstrating additional, active modes of motion [17]. We recently observed a similar phenomenon, by application of fluorescence correlation spectroscopy (FCS) to the measurement of the degree of local chromatin compaction in different stages of the cell cycle and investigation of how this variable regulates the diffusion of small inert molecules [18-20].

Here however, we present a new tracking-based method which can detect chromatin density movement and quantify the mechanical dynamics of chromatin in live cells. The method works by rapidly scanning a line across a fluorescently-labeled chromatin density region to obtain a time series of the chromatin intensity along the line. A Gaussian distribution fit of each scanned line is then performed to generate a track of the center of the chromatin density as a function of time. Autocorrelation analysis on the derived track extracts the characteristic time(s) at which the chromatin density region moves back and forth, and calculation of the track’s centre of mass standard deviation extracts the spatial amplitude of the detected oscillation (in the x-y plane). We use this unconventional approach to particle tracking to study how the mechanical properties of chromatin movement in hESCs are modulated spatiotemporally during differentiation into cardiomyocytes (CM). With this method we show that pluripotency is associated with a highly discrete, energy-dependent frequency of chromatin movement that we refer to as a ‘breathing’ state. We find that this ‘breathing’ state is strictly dependent on the metabolic state of the cell and is progressively silenced during differentiation, thus presumably representing a hallmark of pluripotency maintenance. This is a result that could not have been observed without the nanometer resolution provided by this novel tracking method.

## Results and discussion

### Chromatin dynamics revealed by autocorrelation analysis of a Gaussian track

**Figure 1:**
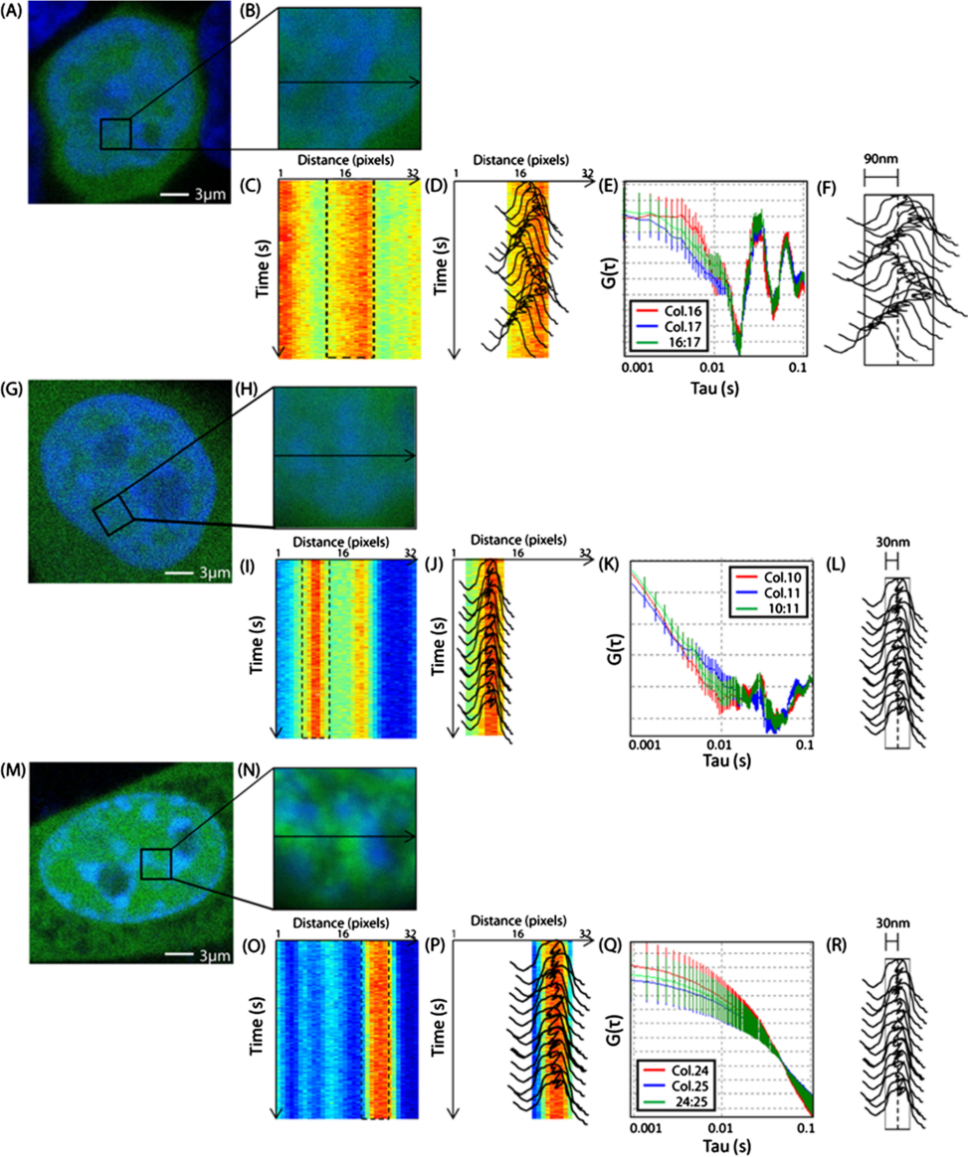
**Extracting the frequency of chromatin movement. **(**A**) hESC nucleus expressing EGFP stained with Hoechst 33342. (**B**) hESC chromatin density region selected for characterization. (**C**) Intensity profile of the line-scan acquired across the hESC chromatin density region as a function of time. (**D**) Schematic of the Gaussian fit and the corresponding track. (**E**) Autocorrelation analysis of the Gaussian track derived for the hESC chromatin density region (columns 13 to 20). The midpoint was defined as column 16 (red curve) and 17 (blue curve). Cross correlation analysis of these two curves (green). (**F**) The track’s center of mass standard deviation in the x-y plane (90 nm). (**G**) HELA nucleus expressing EGFP stained with Hoechst 33342. (**H**) HELA chromatin density region selected for characterization. (**I**) Intensity carpet of the line-scan acquired across the HELA chromatin density region. (**J**) Schematic of the Gaussian fit and the corresponding track. (**K**) Auto- and cross correlation analysis of the Gaussian track derived for the HELA chromatin density region (columns 7 to 13). (**L**) The track’s center of mass standard deviation in the x-y plane (30 nm). (**M**) NIH3T3 nucleus expressing EGFP stained with Hoechst 33342. (**N**) NIH3T3 chromatin density region selected for characterization. (**O**) Intensity carpet of the line-scan acquired across the NIH3T3 chromatin density region. (**P**) Schematic of the Gaussian fit to and the corresponding track. (**Q**) Auto- and cross correlation analysis of the Gaussian track derived for the NIH3T3 chromatin density region (columns 23 to 28). (**R**) The track’s center of mass standard deviation in the x-y plane (30 nm).

To establish our method as sensitive to chromatin density movement, we first compared the mechanical properties of chromatin in pluripotent hESCs, which is known to have an open structure and to be highly dynamic, with the chromatin in fully differentiated cervical cancer HELA cells, where chromatin dynamics are not properly regulated and moderately dynamic, and then with fully differentiated NIH-3 T3 fibroblast cells, where chromatin dynamics are correctly regulated and the chromatin structure is condensed and stable. All cell types were transiently transfected with monomeric EGFP to label the entire volume of the cell, while the nuclei were stained with Hoechst 33342 to visualize the local chromatin density. As can be seen in Figure 1A, a typical hESC nucleus is large, filling almost the entire cell volume, and the chromatin is largely diffuse. For each hESC nucleus selected (N = 10) three regions of chromatin were tested, by zooming in on a single chromatin density region (Figure 1B) and then scanning a line across it rapidly in time (see Experimental Procedures). For each line scan acquired across chromatin, we extract a time series of the Hoechst 33342 intensity (chromatin density) along the line (Figure 1C). As can be seen in Figure 1C the high chromatin density region is positioned approximately in the middle of the line scan (between columns 13 to 20) and appears to be stationary over the duration of the experiment. However, as schematically depicted in Figure 1D, if we fit the columns comprising the chromatin density to a Gaussian distribution function to generate a track of the center of mass of the chromatin density, we find that in fact the chromatin density is moving back and forth. To quantify this chromatin movement, we perform an autocorrelation analysis of the derived track (Figure 1E) and reveal two discrete peaks of positive correlation between 0.01 and 0.1 s, which correspond to the characteristic time(s) at which the chromatin density region shifts back and forth along the line (10 to 100Hz). Calculation of the track’s center of mass standard deviation in the x-y plane reveals the spatial amplitude of the detected oscillation to be 90 to 100 nm (Figure 1F).

If we perform the same experimental procedure across a chromatin density region that originates from a HELA nucleus (unregulated differentiated chromatin), we obtain a slightly different result from that observed in a hESC nucleus (undifferentiated chromatin). As depicted in Figure 1G, a typical HELA nucleus fills approximately 50 to 70% of the cell volume and has a higher overall chromatin content than that observed in an undifferentiated hESC nucleus. As with the hESC experiments, for each HELA nucleus selected (N = 10) we measured chromatin movement in three regions and produced for each (Figure 1H) a time series of the Hoechst 33342 intensity (chromatin density) along the line scan (Figure 1I). As can be seen in Figure 1I, a chromatin density region is positioned between columns 7 and 13 which, similarly to the hESC chromatin density region in Figure 1B, appears to be stationary over the duration of the experiment. As schematically depicted in Figure 1J, if we fit those columns containing the chromatin density region to a Gaussian distribution function and generate a track of the center of mass of the chromatin density region, we find from autocorrelation analysis of the derived track (Figure 1K), that the peaks of positive correlation signifying chromatin vibration in the hESC cell are dampened to almost zero amplitude. This loss of chromatin vibration is accompanied by a reduction in the track’s center of mass standard deviation in the x-y plane to 30 nm (Figure 1L). Thus, the extracted dynamics for differentiated chromatin suggest a more stable and less mobile structure than that observed for hESC undifferentiated chromatin.

If we then again perform the same experimental procedure on a chromatin density region that originates from a NIH3T3 nucleus (regulated differentiated chromatin) we obtain a completely different result to that observed in hESC or HELA nuclei. As depicted in Figure 1M a typical interphase NIH3T3 nucleus fills approximately 30 to 50% of the cell volume and contains very dense regions of heterochromatin inter-dispersed throughout more diffuse euchromatin. As with the hESC and HELA experiments, for each NIH3T3 nucleus selected (N = 10), we measured chromatin movement in three regions and produced for each (Figure 1N) a time series of the Hoechst 33342 intensity (chromatin density) along the line scan (Figure 1O). As can be seen in Figure 1O, a chromatin density region is positioned between columns 23 and 28 which, similarly to the hESC or HELA chromatin density region in Figure 1B and 1H, appears to be stationary over the duration of the experiment. As schematically depicted in Figure 1P, if we fit those columns containing the chromatin density region to a Gaussian distribution function and generate a track of the center of mass of the chromatin density region, we find from autocorrelation analysis of the derived track (Figure 1Q) that there are no peaks of positive correlation; only the decay of a diffusive component of motion between 0.001 and 0.1 s can be measured. Calculation of the track’s center of mass standard deviation in the x-y plane is 30 nm (Figure 1R). Thus the NIH3T3 chromatin behaves like a narrow rope which is shifting in position by random motion, the hESC chromatin actively vibrates back and forth at discrete frequencies that range from 10 to 100Hz and the unregulated differentiated HELA chromatin silences the vibrations characteristic of undifferentiated chromatin to almost no amplitude.

**Figure 2:**
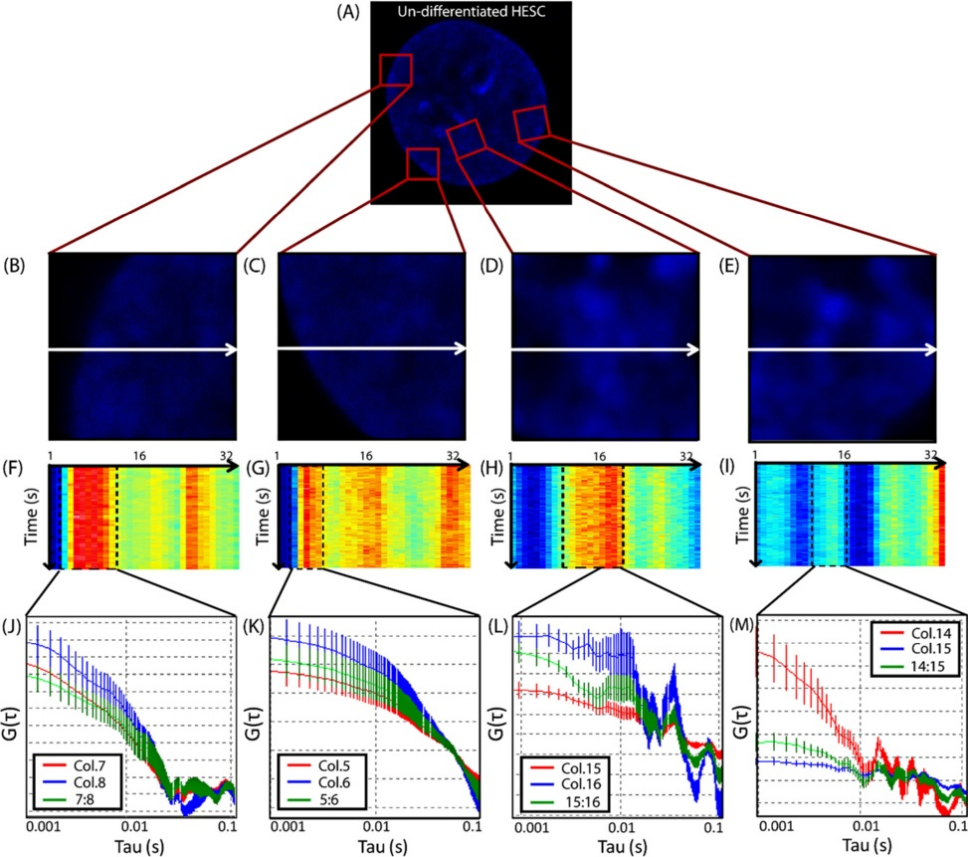
**Control experiment demonstrating that the vibrations detected from undifferentiated human embryonic stem cell (hESC) chromatin are not an artifact of whole cell movement or instrumental vibration.** (**A**) In a single hESC nucleus, (**B**)-(**C**) two line scan experiments were acquired across the nuclear envelope (a structure which should not vibrate and is representative of whole cell movement) and (**D**)-(**E**) two line experiments were acquired across a heterogeneous chromatin region (a structure shown to vibrate in Figure 1A -F). (**F**)-(**I**) The intensity carpet recovered from performing a line scan across each structure depicted in (B)-(E) respectively. (**J**)-(**K**) Auto- and cross correlation analysis of the Gaussian track derived for nuclear envelope movement centered at two adjacent middle columns reveal only the diffusive decay of random motion. (**L**)-(**M**) Auto- and cross correlation analysis of the Gaussian track derived for chromatin density movement centered at two adjacent middle columns, reveal the characteristic discrete peaks of positive correlation which represent different frequency oscillations.

To ensure that the detected vibrations in hESC chromatin were not the result of nuclear or even whole cell movement, we performed control line scan experiments across the nuclear envelope to understand any baseline hESC dynamics. As depicted in Figure 2A, in a single hESC nucleus, two line scan experiments (Figure 2B-C) were acquired across the nuclear envelope (a structure which should not vibrate and is representative of whole cell movement) and two line experiments (Figure 2D-E) were acquired across a heterogeneous chromatin region (a structure shown to vibrate in Figure 1A-F). From application of the same analytical procedure used in Figure 1 to each line scan acquired, we find from autocorrelation of those columns comprising the nuclear envelope (Figure 2F-G) only the diffusive decay of random motion (Figure 2J-K) and, in contrast, for those columns comprising a chromatin density region (Figure 2H-I), discrete peaks of correlation (Figure 2L-M). Thus the different frequency oscillations we detect in Figure 1A-F are not an artifact of whole cell movement or mechanical vibration and do originate from the hESC chromatin.

### Chromatin dynamics and metabolic energy

Based on the results shown in Figure 1E, chromatin seems to oscillate in specific frequency bands. This oscillation could not happen by thermal fluctuations alone; it requires a source of energy. We decided to test whether the hESC chromatin dynamics that we detect vibrating back and forth were energy dependent. To test this hypothesis we depleted the hESC nuclei of ATP and monitored the change in chromatin movement as a function of time. To monitor the extent of ATP depletion and thus the metabolic environment at each time-point during the experiment, we concomitantly measured the local NADH content (which is proportional to the level of ATP present) by the phasor approach to fluorescence lifetime imaging microscopy (FLIM) (for more details see Methods) [21]. Given that this experiment required observation of the hESC nuclei for several hours and the emission properties of NADH and Hoechst 33342 spectrally overlap, we marked the local chromatin density by transient transfection with H2B-EGFP. H2B is reported to be a core histone which does not undergo significant dissociation from the chromatin fiber in the time frame of our experiments, even in the hESC hyperdynamic environment [5]. Thus any movement detected by autocorrelation analysis of a Gaussian track derived from H2B-EGFP can be reasonably ascribed to chromatin movement and not histone dissociation. This is further proved by data in Additional file 1: Figure S1, where we perform concomitant analysis of a chromatin density region marked with Hoechst 33342 (A) and H2B-EGFP (B).

**Figure 3:**
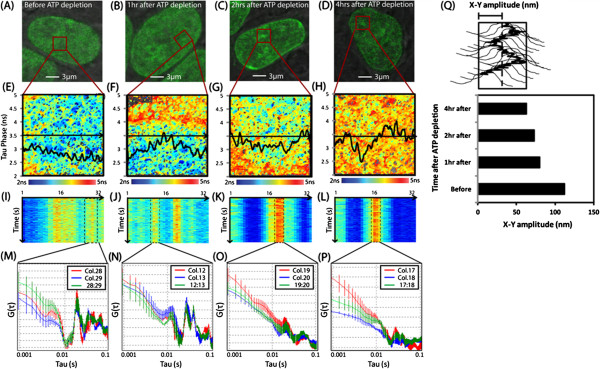
**Energy dependence of human embryonic stem cell (hESC) chromatin movement. **(**A**)-(**D**) A hESC nucleus expressing H2B-GFP before ATP depletion, one hour after ATP depletion, two hours after and four hours after ATP depletion, with the chromatin density region tested from each stage of depletion highlighted by a red box. (**E**)-(**H**) Tau phase map of the free NADH content (scale from 2 ns = high free NADH to 5 ns = low free NADH) that surrounds the selected chromatin density region from different stages of ATP depletion (red box, selected from nuclei (A)-(D)), with the tau phase profile recorded along the actual line scan (black arrow) superimposed. (**I**)-(**L**) Intensity carpet of H2B-EGFP fluorescence along the line scan performed across the chromatin density region from different stages of ATP depletion. (**M**)-(**P**) Auto- and cross correlation analysis of the Gaussian track derived for chromatin movement of the density region centered at two adjacent middle columns, before, one hour, two hours and four hours after ATP depletion. (**Q**) Change in amplitude of chromatin vibration in the X-Y plane upon ATP depletion (N = 5 tracking experiments).

The results of the ATP depletion experiment are depicted in Figure 3. As can be seen in Figure 3A-D, the H2B-GFP localization in the hESC nuclei becomes more compact with increasing time after ATP depletion. The extent of ATP depletion at different time points during the experiment correlates with an incremental reduction in the local free NADH content as indicated by a lengthening of the detected lifetime (tau phase) that surrounds the different chromatin density regions tested (Figure 3E-H). As can be seen in Figure 3I (before ATP depletion), the chromatin density region that is positioned between columns 27 and 30 was fit to a Gaussian distribution to generate a track of chromatin movement that is characteristic of physiological conditions. Autocorrelation analysis of the derived track is depicted in Figure 3M and shows in agreement with Figure 1E, peaks of positive correlation between 0.01 and 0.1 s, that correspond to the frequency (10 to 100Hz) at which the chromatin density region moves back and forth along the line. The track’s standard deviation in the x-y plane at this time reveals the spatial amplitude of this oscillation to be 112 nm. After one hour of ATP depletion, as can be seen in Figure 3N, autocorrelation analysis of chromatin movement still produces peaks of positive correlation between 0.01 and 0.1 s, although with reduced amplitude. The track’s standard deviation in the x-y plane at this time reveals the spatial amplitude of the detected oscillation to be further reduced to 80 nm. After two to four hours of ATP depletion (Figure 3K-O and Figure 3L-P, respectively), autocorrelation analysis of the derived track reveals that the peaks of positive correlation normally seen between 0.01 and 0.1 s are almost extinguished and the spatial amplitude of the detected oscillation to be even further reduced to 63 nm; almost half the original spatial amplitude detected prior to ATP depletion (Figure 3Q). Thus, the observation of hESC chromatin actively vibrating back and forth at discrete frequencies in Figure 1E is an energy dependent phenomenon, given that removal of ATP (observed by reduction of free NADH) gradually dampens the peaks of positive correlation, which were evidence of this mechanical property.

An additional observation that can be drawn from the experiment depicted in Figure 3 is that, if by depleting ATP we concomitantly deplete free NADH, then potentially the reduction in chromatin movement detected is an indirect result of the removal of free NADH; a metabolite that has been proven to play a role in DNA compaction regulation and differentiation (see next section and discussion). For example, pluripotent stem cells which have an open and dynamic chromatin structure are known to have higher levels of free NADH in the nucleus than differentiated stem cells which have a more compact chromatin structure. In part, this is because high levels of NAD^+^ can lead to the activation of HDACs of the sirtuin class, which induce deacetylation of histones and thus chromatin compaction [22]. Given this relationship, the ATP depletion experiment depicted in Figure 3 potentially mimicked the changes in chromatin structure experienced by hESC nuclei upon differentiation.

### Chromatin dynamics and differentiation

**Figure 4:**
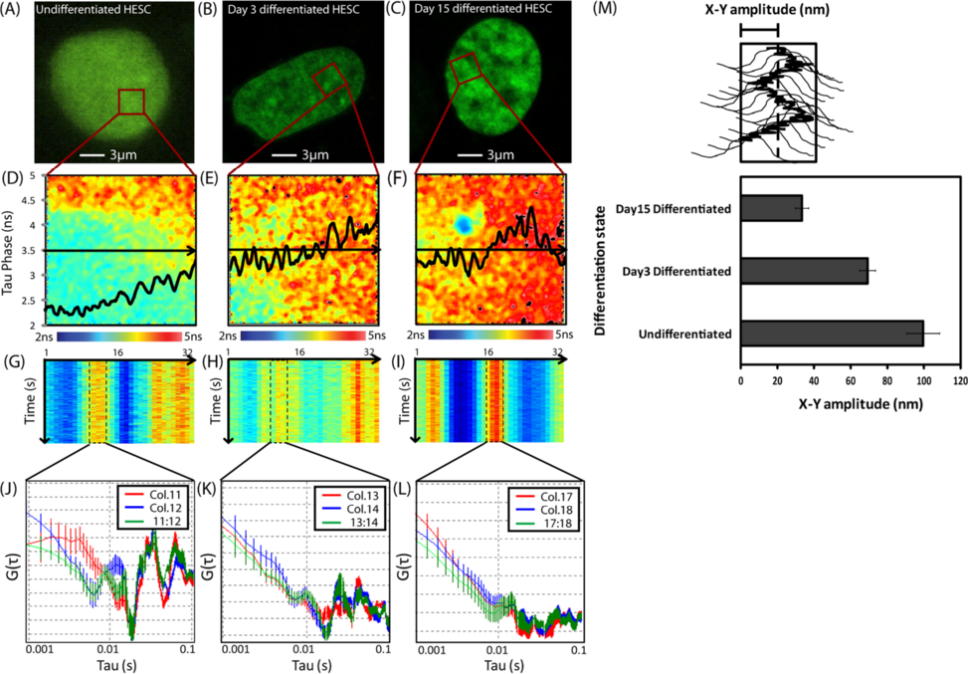
**Changes in human embryonic stem cell (hESC) chromatin movement upon differentiation. **(**A**)-(**C**) Undifferentiated, day-3 differentiated and day-15 differentiated ESC nucleus expressing H2B-GFP, with the chromatin density region tested from each stage of differentiation highlighted by a red box. (**D**)-(**F**) Tau phase map of the free NADH content (scale from 2 ns = high free NADH to 5 ns = low free NADH) that surrounds the selected chromatin density region from different stages of differentiation (red box, selected from nuclei (A)**-**(C)), with the tau phase profile recorded along the actual line scan (black arrow) superimposed. (**G**)-(**I**) Intensity carpet of H2B-EGFP fluorescence along the line scan performed across the chromatin density region from the different stages of differentiation. (**J**)-(**L**) Auto- and cross correlation analysis of the Gaussian track derived for chromatin movement of the density region centered at two adjacent middle columns in undifferentiated, day-3 differentiated and day-15 differentiated ESC nuclei. (**M**) Change in amplitude of chromatin vibration in the X-Y plane upon differentiation (N = 5 tracking experiments).

We showed that an active ‘breathing’ behavior characterizes the chromatin movement in hESCs while it is absent in truly differentiated NIH3T3 fibroblasts. If differentiation plays a role in silencing the ‘breathing’ chromatin dynamics, then we should be able to prove it in properly designed experiments. To this aim, we applied our established tracking method to measure and compare chromatin movement in undifferentiated hESCs with day-3 differentiated hESCs and day-15 differentiated hESCs (cardiomyocytes). The results of this experiment are depicted in Figure 4. As can be seen from Figure 4A-C, the H2B-GFP localization in the hESC nuclei is very diffuse and then, upon differentiation, becomes increasingly more compact. As depicted in Figure 4D, the local free NADH content that surrounds the chromatin density region tested within the undifferentiated hESC nucleus is high (short tau phase) and then gradually decreases going from day-3 differentiation (Figure 4E) to day-15 (Figure 4F). As previously observed for undifferentiated hESC chromatin, autocorrelation analysis of the Gaussian track for the chromatin density region positioned between columns 9 and 13 in Figure 4G produces peaks of positive correlation between 0.01 and 0.1 s (Figure 4J). By contrast, the same analysis performed on day-3 differentiated hESC chromatin results in dampened peaks of correlation between 0.01 and 0.1 s (Figure 4H-K). Remarkably, those peaks are completely extinguished in day-15 differentiated hESC chromatin density regions (Figure 4I-K). This gradual dampening of chromatin fiber movement observed upon differentiation is accompanied by a gradual reduction in the spatial amplitude of the detected vibration, from 87 nm to 32 nm (as depicted in Figure 4M).

## Conclusions

Local chromatin movements are a prerequisite for changing the transcriptional status of a gene, whether for repression or activation [23]. However, how these chromatin movements can result in a dense chromatin structure being opened locally to allow access to its genes remains unresolved. It is known that many essential proteins that interact with DNA do not have access to DNA when it is wrapped, and even the unwrapped sections are somewhat buried inside the dense 30 nm fiber. The cell therefore must have mechanisms at hand to open (unfold) the fiber and then, somehow, to unwrap the DNA [24]. Given that chromatin structural changes involve length scales of many orders of magnitude (from ångströms to micrometers) and dynamic processes that take place over a wide range of timescales, measuring a complete ‘picture’ of chromatin dynamics can be difficult [25].

Standard confocal microscopy has the capability to measure chromatin structure in live cells only at the 200 nanometer and second scale resolution. Here, we increase the confocal spatiotemporal range and present a new tracking method to measure chromatin mechanical dynamics in live cells on the 10 nanometer and micro- to millisecond timescale. From acquisition of a line scan measurement across a fluorescently labeled chromatin density region, we generate a track of the center of mass of the chromatin density region as a function of time by a Gaussian distribution fit. The autocorrelation analysis of the derived track yields a quantitative description of the temporal dynamics of the local chromatin movement, whilst calculation of the track’s center of mass standard deviation in the x-y plane, reveals the spatial amplitude of the detected oscillation with nanometer resolution. From application of this method to hESCs we find undifferentiated hESC chromatin to vibrate at specific frequency bands (10 to 100Hz), a phenomenon we proved to be dependent on the metabolic state of the cell and progressively silenced during differentiation. We thus propose that the measured chromatin vibrations in hESCs may represent a hallmark of pluripotency which serve as a mechanism to maintain the genome in a transcriptionally accessible state.

Furthermore, given that the energy dependent vibrations detected from hESC chromatin occur on a timescale comparable to known micro- to millisecond fluctuations in nucleosome structure [26], we propose this mechanical movement to be one of the mechanisms ‘at hand’ to the hESC genome that maintains the chromatin in an open (unfolded) conformation. It has been shown through competitive protein binding to nucleosomal DNA that thermal fluctuations lead to a partial unwrapping of the DNA from the nucleosome, providing intermittent access to nucleosomal DNA. However in order to gain direct access to DNA (unwrap the fiber) the assistance of chromatin-remodelling complexes is required. These are large multiprotein complexes that use energy by burning ATP [27,28]. This picture reminds us of the observations made by Meshorer *et al*. on protein hyperdynamic binding to hESC chromatin (see Background), which contributes to the maintenance of chromatin in a globally relatively open, ‘breathing’ state. This intriguing scenario will guide future experiments to elucidate the physical origin of observed differences in the chromatin dynamic state between embryonic and fully differentiated cells.

## Methods

### Cell culture and treatments

Feeder-independent H9 hESCs (WiCELL, Wisconsin, USA, http://www.wicell.org) were maintained in mTeSR1 medium (StemCell Technologies, Canada, http://www.stemcell.com) on tissue culture plates coated with Matrigel (BD Biosciences, California, USA, http://www.bdbiosciences.com). Cell culture medium was exchanged daily, and cells were passaged once they reached 80 to 90% confluence. Two days before the undifferentiated hESC experiment, cells were detached using Accutase (Innovative Cell Technologies, California, USA, http://www.innovativecelltech.com) and transferred to a MatTek 35-mm glass bottom dishes coated with Matrigel. ROCKi (Reagent Direct, California, USA, http://www.reagentsdirect.com) was added to enhance the viability of the cells. A day before the experiment, hESCs were transiently transfected with a H2B-EGFP plasmid using Lipofectamine 2000 according to the manufacturer’s protocol. Energy depletion experiments were conducted by using sodium azide and 2-deoxy-d-glucose, as described elsewhere [29].

To differentiate the cells, a slight modification of directed cardiac differentiation method was used as previously described [30]. Briefly, undifferentiated hESCs maintained on Matrigel in mTeSR1 medium were dissociated with Accutase solution. Cells were replated to glass bottom petri-dishes, and allowed to expand to confluence (approximately 3 days) in mTeSR1 medium. Once hESCs reached confluence, mTeSR1 medium was replaced with RPMI-B27 medium (Invitrogen, New York, USA, http://www.invitrogen.com) supplemented with 100 ng/mL human recombinant activin A (R&D Systems, Minneapolis, USA, http://www.rndsystems.com) for 24 hours. This is now day 0 of differentiation. After 24 hours, or on day 1 of the differentiation, the culture medium was replaced with RPMI-B27 medium supplemented with 10 ng/mL human recombinant BMP4 and 10 ng/mL human recombinant bFGF (R&D Systems, Minneapolis, USA, http://www.rndsystems.com) for 4 days. On day 5 of differentiation, the culture medium was replaced with RPMI-B27 medium without supplements, and medium was exchanged every 3 days thereafter. On day 3 of the differentiation, 70% of the cells are in the mesoderm lineage as indicated by T/Brachyury stain (as confirmed by FACS analysis, data not shown), and on day 15, 75% of the cells are cardiomyocytes as indicated by α-MHC stain (as confirmed by FACS analysis, data not shown). Depletion of metabolic energy assay was performed following an established protocol [29].

### Microscopy

The microscopy measurements were performed on a Zeiss LSM710 Quasar laser scanning microscope, using a 40X water immersion objective 1.2 N.A. (Zeiss, Germany, http://www.zeiss.com/microscopy). EGFP was excited with the 488 nm emission line of an Argon laser and Hoechst 33342 was excited with a 405 nm diode laser. Detailed description of the experimental settings used for the line-scan measurement is present in a previous publication [19]. Briefly, we acquire data by rapidly scanning a diffraction limited laser beam (488 nm) along a line drawn inside the nucleus across a discontinuity in chromatin density. Measuring a line of 32 pixels at maximum zoom, we sample fluorescence every 100 nm and this results in a line length of 5.14 μm. The maximum scanning speed for these settings was selected (pixel dwell time 6.3 μs, line time 0.472 ms) so that the fluorescent molecules could be correlated in time between lines. In general for each experiment, 200,000 consecutive lines (with no intervals between lines) were acquired. Time regions within each experiment (typically approximately 64,000 lines, corresponding to approximately 30s) with no average change in fluorescence intensity (for example, photo-bleaching) were then selected for the correlation analysis.

For the NADH experiments, the FLIM data were also acquired with the Zeiss LSM710 Quasar laser scanning microscope which is coupled to a 2-photon Ti:Sapphire laser (Spectra-Physics Mai Tai, Newport Beach, USA, http://www.newport.com/cms/brands/spectra-physics) producing 120 fs pulses at a repetition of 80 MHz, and a ISS A320 FastFLIM box. NADH was excited at 740 nm with the 2-photon laser: this wavelength caused negligible excitation of the H2B-EGFP. A SP 610 nm dichroic filter was used to separate the fluorescence signal from the laser light in the LSM710. The fluorescence signal was directed through a 495 LP NADH/FAD filter, and the signal split between two photomultiplier detectors (H7422P-40 of Hamamatsu*,*http://www.hamamatsu.com), with the following bandwidth filters in front of each: NADH 460/80 and FAD 540/50, respectively. Only the NADH channel was collected. For image acquisition, the frame size was set to 256 x 256 pixels and the pixel dwell time to 25.61 μs/pixel. The average laser power at the sample was maintained at the mW level. Calibration of the system and phasor plot analysis was performed by measuring fluorescein (pH 9.0), which has a known single exponential lifetime of 4.04 ns.

### Data analysis

Calculation of Gaussian track, auto-correlation function (ACF) and standard deviation of the tracks center of mass was done using the SimFCS software developed at the Laboratory for Fluorescence Dynamics (http://www.lfd.uci.edu) [18,19,31,32]. Intensity data are presented by using a carpet representation in which the *x*-coordinate corresponds to the point along the line (pixels) and the *y*-coordinate corresponds to the time of acquisition. Every 50 lines within the intensity carpet are then averaged and the columns containing a high density chromatin region fit to a Gaussian distribution. The center of mass of this distribution is then recorded and a track generated as a function of time. Autocorrelation analysis of the derived track is then performed using the sampling frequency determined by the line scan time. By plotting the ACF on a log scale, we are able to detect the characteristic time(s) at which the chromatin density region moves back and forth from a millisecond to second timescale. Calculation of the track’s center of mass standard deviation reveals the spatial amplitude of chromatin fiber movement back and forth with nanometer resolution. To ensure we were extracting mechanical dynamics representative of the chromatin density region and not of a single column, we defined the high density chromatin region by selecting two adjacent middle columns with a half width that took into account the entire structure and then generating two tracks of the center of mass of these two distributions. This then allowed us to perform cross correlation analysis between the two tracks and thus conclude that the two selected columns were moving together as a part of a structure.

The phasor transformation and data analysis of the NADH experiments were also performed using the SimFCS software, as described in previously published papers [21,33]. Briefly, the phasor approach to FLIM transforms the fluorescence decay histogram at each pixel in an image into the sine and cosine components which are then represented in a two dimensional polar plot (phasor plot). Each pixel of an image therefore gives rise to a single point (phasor) in the phasor plot and when used in reciprocal mode, enables each point of the phasor plot to be mapped to each pixel of the image [33]. Since phasors follow simple vector algebra, it is possible to determine the fractional contribution of two or more independent molecular species coexisting in the same pixel. In the case of two species, all possible weightings give a phasor distribution along a linear trajectory that joins the phasors of the individual species in pure form.

## Abbreviations

ESC: Embryonic stem cell; hESC: Human embryonic stem cell; CM: Cardiomyocyte; ATP: Adenosine-5^′^-triphosphate; FCS: Fluorescence correlation spectroscopy; EGFP: Enhanced green fluorescent protein; H2B: Histone H2B; NADH: Nicotinamide adenine dinucleotide; FLIM: Fluorescence lifetime imaging microscopy.

## Competing interests

The authors declare that they have no competing interests.

## Authors’ contributions

EH carried out the experiments and data analysis, designed the research and wrote the paper. FC designed the research and wrote the paper. AC carried out cell culture and sample preparation. MK provided the hESCs along with discussions and input regarding their maintenance and preparation. EG designed the research and in algorithms for chromatin tracking. All authors read and approved the final manuscript.

## Supplementary Material

Additional file 1 **Figure S1.** Comparison of the chromatin dynamics recovered from marking DNA with Hoechst 33342 versus transient transfection with H2B-EGFP. (**A**)-(**B**) Chromatin density region within a hESC nucleus that is both stained with Hoechst 33342 and expressing H2B-EGFP, respectively. (**C**)-(**D**) Intensity profile of the Hoechst 33342 stain and H2B-EGFP fluorescence along the selected line scan. (**E**)-(**F**) Intensity carpet of the line scan acquired across the hESC chromatin density region in the Hoechst 33342 channel (blue) and H2B-EGFP channel (green), respectively. (**G**)-(**H**) Autocorrelation analysis of the Gaussian track derived for the selected hESC chromatin density region movement, as detected from the Hoechst 33342 and H2B-EGFP fluorescence, respectively. As can be seen from comparison of (G) with (H) the positive peaks of correlation detected between 0.1 and 1 s agree between the two channels. Click here for file

## References

[B1] O'Shea KS Self-renewal versus differentiation of mouse embryonic stem cells Biol Reprod 2004 71 1755 1765 10.1095/biolreprod.104.028100 15329329

[B2] Meshorer E Misteli T Chromatin in pluripotent embryonic stem cells and differentiation Nat Rev Mol Cell Biol 2006 7 540 546 10.1038/nrm1938 16723974

[B3] Mattout A Meshorer E Chromatin plasticity and genome organization in pluripotent embryonic stem cells Curr Opin Cell Biol 2010 22 334 341 10.1016/j.ceb.2010.02.001 20226651

[B4] Meshorer E Chromatin in embryonic stem cell neuronal differentiation Histol Histopathol 2007 22 311 319 17163405 10.14670/HH-22.311

[B5] Meshorer E Hyperdynamic plasticity of chromatin proteins in pluripotent embryonic stem cells Dev Cell 2006 10 105 116 10.1016/j.devcel.2005.10.017 16399082 PMC1868458

[B6] Meshorer E Imaging chromatin in embryonic stem cells StemBook (ed. The Stem Cell Research Community) 2008 StemBook http://www.stembook.org 20614626

[B7] Park SH Ultrastructure of human embryonic stem cells and spontaneous and retinoic acid-induced differentiating cells Ultrastruct Pathol 2004 28 229 238 10.1080/01913120490515595 15693634

[B8] Cammas F Cell differentiation induces TIF1beta association with centromeric heterochromatin via an HP1 interaction J Cell Sci 2002 115 3439 3448 12154074 10.1242/jcs.115.17.3439

[B9] Kurisaki A Chromatin-related proteins in pluripotent mouse embryonic stem cells are downregulated after removal of leukemia inhibitory factor Biochem Biophys Res Commun 2005 335 667 675 10.1016/j.bbrc.2005.07.128 16099433

[B10] Bultman S A Brg1 null mutation in the mouse reveals functional differences among mammalian SWI/SNF complexes Mol Cell 2000 6 1287 1295 10.1016/S1097-2765(00)00127-1 11163203

[B11] Klochendler-Yeivin A The murine SNF5/INI1 chromatin remodeling factor is essential for embryonic development and tumor suppression EMBO Rep 2000 1 500 506 11263494 10.1093/embo-reports/kvd129 PMC1083796

[B12] Cao S The high-mobility-group box protein SSRP1/T160 is essential for cell viability in day 3.5 mouse embryos Mol Cell Biol 2003 23 5301 5307 10.1128/MCB.23.15.5301-5307.2003 12861016 PMC165710

[B13] Stopka T Skoultchi AI The ISWI ATPase Snf2h is required for early mouse development Proc Natl Acad Sci USA 2003 100 14097 14102 10.1073/pnas.2336105100 14617767 PMC283552

[B14] Robinett CC In vivo localization of DNA sequences and visualization of large-scale chromatin organization using lac operator/repressor recognition J Cell Biol 1996 135 1685 1700 10.1083/jcb.135.6.1685 8991083 PMC2133976

[B15] Marshall WF Interphase chromosomes undergo constrained diffusional motion in living cells Curr Biol 1997 7 930 939 10.1016/S0960-9822(06)00412-X 9382846

[B16] Chubb JR Boyle S Perry P Bickmore WA Chromatin motion is constrained by association with nuclear compartments in human cells Current biology: CB 2002 12 439 445 10.1016/S0960-9822(02)00695-4 11909528

[B17] Levi V Ruan Q Plutz M Belmont AS Gratton E Chromatin dynamics in interphase cells revealed by tracking in a two-photon excitation microscope Biophys J 2005 89 4275 4285 10.1529/biophysj.105.066670 16150965 PMC1366992

[B18] Hinde E The impact of mitotic versus interphase chromatin architecture on the molecular flow of EGFP by pair correlation analysis Biophys J 2011 100 1829 1836 10.1016/j.bpj.2011.02.024 21463597 PMC3072664

[B19] Hinde E Cardarelli F Digman MA Gratton E In vivo pair correlation analysis of EGFP intranuclear diffusion reveals DNA-dependent molecular flow Proc Natl Acad Sci USA. Proceedings of the National Academy of Sciences of the United States of America 2010 107 16560 16565 10.1073/pnas.1006731107 PMC2944750 20823232

[B20] Hinde E Cardarelli F Digman MA Gratton E Changes in chromatin compaction during the cell cycle revealed by micrometer-scale measurement of molecular flow in the nucleus Biophys J 2012 102 691 697 10.1016/j.bpj.2011.11.4026 22325293 PMC3274830

[B21] Stringari C Phasor approach to fluorescence lifetime microscopy distinguishes different metabolic states of germ cells in a live tissue Proc Natl Acad Sci USA 2011 108 13582 13587 10.1073/pnas.1108161108 21808026 PMC3158156

[B22] Katada S Imhof A Sassone-Corsi P Connecting Threads: epigenetics and metabolism Cell 2012 148 24 28 10.1016/j.cell.2012.01.001 22265398

[B23] Litt MD Simpson M Gaszner M Allis CD Felsenfeld G Correlation between histone lysine methylation and developmental changes at the chicken beta-globin locus Science 2001 293 2453 2455 10.1126/science.1064413 11498546

[B24] Workman JL Kingston RE Alteration of nucleosome structure as a mechanism of transcriptional regulation Annu Rev Biochem 1998 67 545 579 10.1146/annurev.biochem.67.1.545 9759497

[B25] Schiessel H The physics of chromatin J Phys Condens Matter 2003 15 R699 R774 10.1088/0953-8984/15/19/203 25563698

[B26] Widom J Structure, dynamics, and function of chromatin in vitro Annu Rev Biophys Biomol Struct 1998 27 285 327 10.1146/annurev.biophys.27.1.285 9646870

[B27] Flaus A Owen-Hughes T Mechanisms for ATP-dependent chromatin remodeling Curr Opin Genet Dev 2001 11 148 154 10.1016/S0959-437X(00)00172-6 11250137

[B28] Kornberg RD Lorch Y Twenty-five years of the nucleosome, fundamental particle of the eukaryote chromosome Cell 1999 98 285 294 10.1016/S0092-8674(00)81958-3 10458604

[B29] Cardarelli F Serresi M Bizzarri R Giacca M Beltram F In vivo study of HIV-1 Tat arginine-rich motif unveils its transport properties Mol Ther 2007 15 1313 1322 10.1038/sj.mt.6300172 17505482

[B30] Laflamme MA Cardiomyocytes derived from human embryonic stem cells in pro-survival factors enhance function of infarcted rat hearts Nat Biotechnol 2007 25 1015 1024 10.1038/nbt1327 17721512

[B31] Cardarelli F Gratton E In vivo imaging of single-molecule translocation through nuclear pore complexes by pair correlation functions PLoS One 2010 5 10.1371/journal.pone.0010475 PMC2862743 20454622

[B32] Digman MA Gratton E Imaging barriers to diffusion by pair correlation functions Biophys J 2009 97 665 73 10.1016/j.bpj.2009.04.048 19619481 PMC2711318

[B33] Digman MA Caiolfa VR Zamai M Gratton E The phasor approach to fluorescence lifetime imaging analysis Biophys J 2008 94 2 L14 L16 10.1529/biophysj.107.120154 17981902 PMC2157251

