# Supplementary figures and images for: Tracking the mechanical dynamics of human embryonic stem cell chromatin
Source: Epigenetics Chromatin. 2012 Dec 21;5:20. doi: 10.1186/1756-8935-5-20 (PMC3570407; doi:10.1186/1756-8935-5-20)

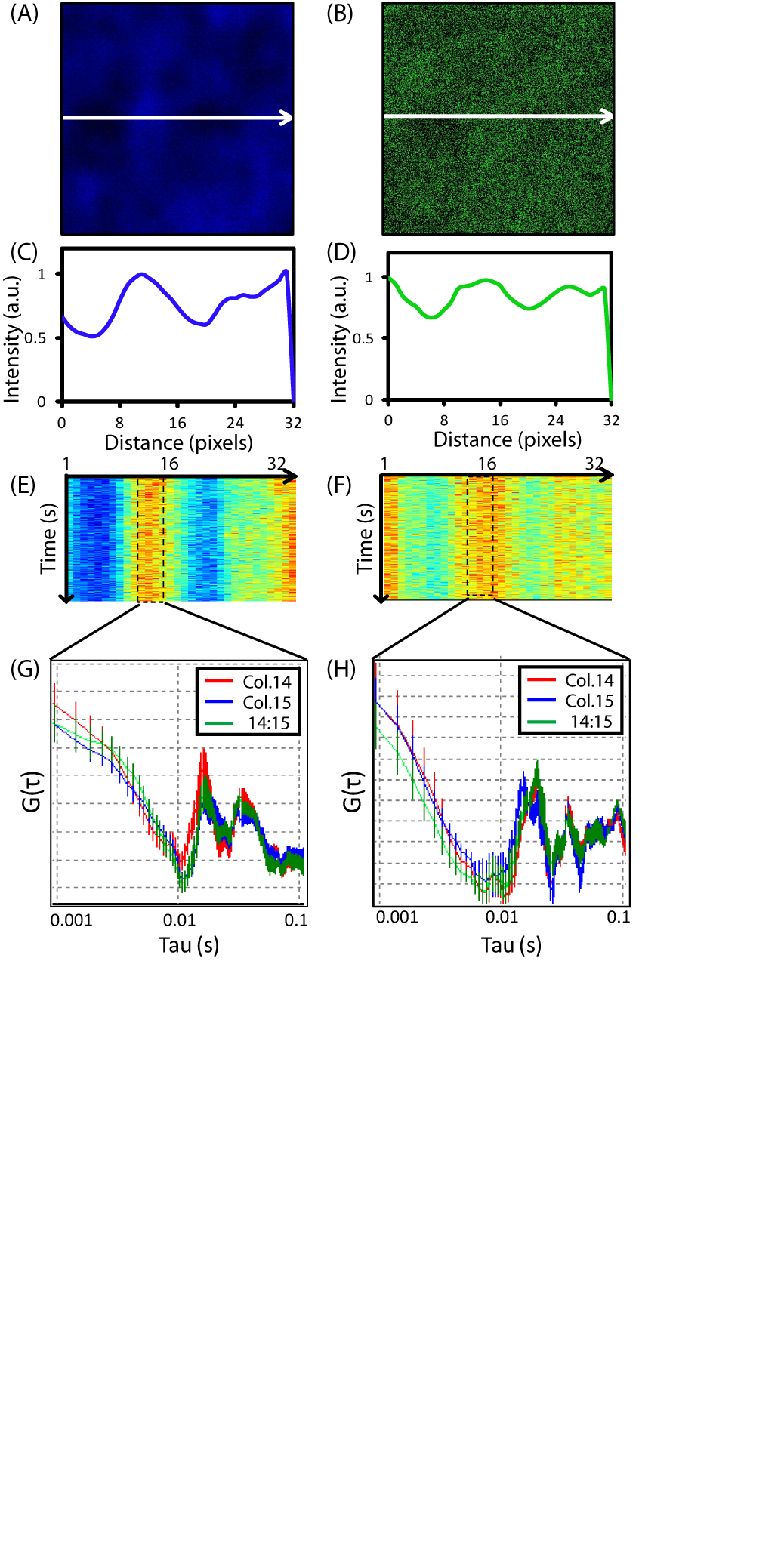

Supplement: Additional file 1 — Figure S1. Comparison of the chromatin dynamics recovered from marking DNA with Hoechst 33342 versus transient transfection with H2B-EGFP. (A)-(B) Chromatin density region within a hESC nucleus that is both stained with Hoechst 33342 and expressing H2B-EGFP, respectively. (C)-(D) Intensity profile of the Hoechst 33342 stain and H2B-EGFP fluorescence along the selected line scan. (E)-(F) Intensity carpet of the line scan acquired across the hESC chromatin density region in the Hoechst 33342 channel (blue) and H2B-EGFP channel (green), respectively. (G)-(H) Autocorrelation analysis of the Gaussian track derived for the selected hESC chromatin density region movement, as detected from the Hoechst 33342 and H2B-EGFP fluorescence, respectively. As can be seen from comparison of (G) with (H) the positive peaks of correlation detected between 0.1 and 1 s agree between the two channels. [file 1756-8935-5-20-S1.tiff]
